# Aging trajectories and functional capacity: insights from the Otassha study

**DOI:** 10.1007/s40520-024-02803-w

**Published:** 2024-07-16

**Authors:** Nicola Veronese

**Affiliations:** Department of Internal Medicine and Geriatrics, Geriatric Unit, University of Palermo, Via del Vespro, 141, Palermo, 90127 Italy

The global demographic shift towards an aging population has spurred a wealth of research aimed at understanding how aging affects functional capacity among older adults [1]. One notable contribution to this field is the Otassha study, which focuses on aging trajectories in higher-level functional capacity among community-dwelling older Japanese adults [2]. This study offers crucial insights into how different aspects of functional capacity evolve with age, informing both healthcare practice and policy aimed at promoting healthy aging in a population characterized by a long life-expectancy.

The Otassha study meticulously examines the aging trajectories of various subscales in higher-level functional capacity, a crucial determinant of independence and quality of life in older adults [2]. Higher-level functional capacity encompasses abilities beyond basic activities of daily living, such as managing finances, using transportation, and engaging in social and recreational activities. These abilities are vital for maintaining autonomy and active participation in society, and their decline can have significant implications for individuals and the broader community.

The study identifies several key subscales of higher-level functional capacity, including intellectual activity, social role, and instrumental activity of daily living (IADL). By analyzing these subscales, the Otassha study provides a nuanced understanding of how different facets of functional capacity decline at varying rates. This is crucial, as it underscores that aging is not a monolithic process; rather, it affects different domains of functioning in distinct ways.

One of the pivotal findings of the Otassha study is the differential aging trajectories observed across these subscales. Intellectual activities, for instance, appear to decline more gradually compared to physical IADLs. This suggests that cognitive engagement and mental stimulation might be sustained longer in the aging process, potentially offering a buffer against the overall decline in functional capacity. The implications for intervention are profound: promoting cognitive activities such as reading, puzzles, and social engagement could help mitigate some of the declines associated with aging.

In contrast, the steeper decline observed in physical IADLs highlights the vulnerability of physical functioning in the aging population. This reinforces the importance of regular physical activity and interventions aimed at maintaining mobility and strength. Tailored exercise programs, fall prevention strategies, and rehabilitation services become critical in supporting older adults to retain their independence and reduce the risk of disability [3]. 

The social role subscale also presents intriguing findings. Social engagement, an essential component of healthy aging, shows a complex trajectory influenced by a variety of factors including health status, mobility, and social networks [4]. The Otassha study reveals that while social roles may diminish with age, maintaining social connections and participating in community activities can significantly bolster overall functional capacity. This highlights the necessity of fostering social environments that encourage interaction and provide opportunities for older adults to contribute meaningfully to their communities.

Importantly, the Otassha study also sheds light on gender differences in aging trajectories. Women, on average, live longer than men but often experience higher rates of disability and functional decline [2]. The study’s findings suggest that gender-specific interventions may be warranted to address the unique challenges faced by older women, such as tailored physical activity programs and support networks that cater to their needs.

From a policy perspective, I believe that the Otassha study’s insights are invaluable. As nations grapple with the economic and social implications of an aging population, evidence-based policies become essential. Governments and health organizations must prioritize initiatives that promote higher-level functional capacity, such as funding for community centers, accessible transportation, and age-friendly urban planning. Additionally, policies that support lifelong learning and mental stimulation can help sustain intellectual activities and delay cognitive decline.

Healthcare systems, too, must adapt to the changing needs of an aging population. Integrating geriatric care and multidisciplinary approaches into primary care can ensure that older adults receive comprehensive assessments and tailored interventions, such as comprehensive geriatric assessment [5]. Training healthcare professionals in geriatric principles and creating supportive environments for caregivers are crucial steps in enhancing the quality of care for older adults.

In conclusion, I think that the Otassha study provides a comprehensive analysis of aging trajectories in higher-level functional capacity among community-dwelling older Japanese adults. Its findings underscore the importance of a multifaceted approach to aging, one that addresses the distinct trajectories of intellectual, physical, and social functioning. By implementing targeted interventions and supportive policies, we can enhance the functional capacity of older adults, enabling them to lead fulfilling, independent lives well into their later years. This not only improves individual well-being but also contributes to the resilience and sustainability of our aging societies.
